# NRP1 regulates autophagy and proliferation of gastric cancer through Wnt/β-catenin signaling pathway

**DOI:** 10.18632/aging.204560

**Published:** 2023-03-07

**Authors:** Qi-Ying Yu, Yue Han, Jia-Hui Lu, Yan-Jie Sun, Xing-Hua Liao

**Affiliations:** 1 Institute of Biology and Medicine, College of Life and Health Sciences, Wuhan University of Science and Technology, Wuhan 430081, Hubei, P.R. China; 2 Jinan People’s Hospital Affiliated to Shandong First Medical University, Shandong, Jinan City People’s Hospital, Jinan 271199, Shandong, P.R. China; 3 Beidahuang Group General Hospital, Heilongjiang Province Second Cancer Hospital, Harbin 150000, Heilongjiang, P.R. China

**Keywords:** autophagy, gastric cancer, NRP1

## Abstract

Gastric cancer possesses high lethality rate, and its complex molecular mechanisms of pathogenesis lead to irrational treatment outcomes. Autophagy plays a dual role in cancer by both promoting and suppressing the cancer. However, the role of autophagy in gastric cancer is still vague. Therefore, in this study, we first obtained autophagy-related genes from the Human Autophagy Database, and then applied consensus clustering analysis to analyse the molecular subtypes of gastric cancer samples in the TCGA database. The genes obtained after subtyping were then applied to construct risk prognostic model. Following this, PCA and tSNE assessed risk scores with good discriminatory ability for gastric cancer samples. The results of Cox regression analysis and time-dependent ROC curve analysis indicated that the model had good risk prediction ability. Finally, NRP1 was selected as the final study subject in the context of expression pairwise analysis, Kaplan-Meier curves and external validation of the GEO dataset. *In vitro* experiments showed that NRP1 has the ability to regulate the proliferation and autophagy of gastric cancer cells by affecting the Wnt/β-catenin signalling pathway. Similarly, *in vivo* experiments have shown that NRP1 can affect tumour growth *in vivo*. We therefore propose that NRP1 can be used as both a prognostic factor and a therapeutic target through the regulation of autophagy in gastric cancer.

## INTRODUCTION

Gastric cancer is an aggressive disease of the digestive system which possesses the fifth highest diagnosis rate and the third highest lethality rate [1]. Currently, the treatment strategies for gastric cancer include immunotherapy, surgical excision, chemotherapy, radiotherapy and etc. Although these strategies have made great progress, the overall curative effect of gastric cancer patients is still not satisfactory [2]. The reason for this situation is that there are no obvious symptoms in the early stages of the patient, and advanced gastric cancer patients are at risk of metastasis and recurrence after surgical resection or other treatments [3, 4]. Therefore, it is more realistic to further study the pathogenesis and screen effective risk prognostic factors in order to enhance the precise therapeutic outcome in gastric cancer.

Autophagy is a finely regulated cellular metabolic mechanism, which can phagocytose and digest biomacromolecules or damaged organelles in cells to provide energy and protection for cells to ensure favorable survival conditions in the face of insufficient nutrition or adverse surrounding environment [5, 6]. Autophagy is classified as microautophagy, macroautophagy and molecular chaperone-mediated autophagy depending on the method of cargo capture [7]. Interestingly, however, autophagy plays a dual role in complex regulation, acting as both a suppressor and promoter of cancer. Abnormal autophagy can also cause cell death called type II programmed cell death, which is different from apoptosis, ferroptosis and pyroptosis [8, 9]. And, in recent years, an increasing number of studies report that autophagy is involved in the proliferation, metastasis, invasion and drug resistance of gastric cancer [10–12].

Since autophagy plays an important role in gastric cancer, the research on the autophagy related genes and the corresponding treatment around these genes has become a hot topic in recent years. Therefore, we reasonably hypothesised that it would be of great value to use autophagy related genes to construct a risk prognostic model and to use prognostic genes as therapeutic research targets.

In this study, we first extracted the expression data of autophagy-related genes for differential expression analysis and then performed consensus clustering analysis to obtain a broader perspective of the study. Based on the results of the consensus clustering analysis, we classified the gastric cancer patient samples into two subtypes (C1 and C2). LASSO regression analysis was then used to screen the autophagy-related genes from the consensus clustering analysis and to construct a risk-prognosis model. Lastly, with the validation and screening of the GEO datasets GSE26899, GSE29272, GSE118916 and GSE54129, we selected NRP1 from the 12 risk genes as the target for the subsequent study. After experiments, the results showed that NRP1 regulates the proliferation and autophagy of gastric cancer cells through the Wnt/β-catenin signaling pathway. In summary, our results indicate that we have established a validated effective autophagy gene-related risk prognostic model and provide a promising therapeutic target, NRP1.

## MATERIALS AND METHODS

### EdU assay

Cells were cultured on a 14mm cell culture coverslide at 37° C with 5% CO_2_ overnight. Then complete medium containing 50 mM EdU was added and incubated for 2 hours. Finally, reagents were added sequentially according to the instructions and EdU positive cells were observed by laser confocal microscopy and photographed.

### Cellular immunofluorescence

Cells in knockdown and control group were inoculated on the cover slide and cultured overnight at 37° C and 5% CO_2_. On the second day, we took out the well plates and fixed the cells with 4% paraformaldehyde. Then Triton-X100 was added to permeate the cell membrane and PBS containing 1%BSA blocked the nonspecific antigen at 25° C for 1 hour. Anti-Ki67 primary antibody working solution was added and incubated at 4° C overnight (Abclonal, China). After PBS washing, FITC-labeled secondary antibody was added and incubated for 1 hour at 25° C (Abclonal, China). Finally, the expression of Ki67 was observed by confocal laser microscope and photographed.

### Construction of xenograft tumor model

1x10^7^ cells from different groups were injected subcutaneously into the back of 3-week-old male nude mice. After that, tumor volume was measured every 6 days and tumor volume growth curve was plotted [13]. On the 18th day of incubation, animal imaging was performed and mice were sacrificed for tumor stripping and weighing.

### Autophagy assay

Firstly, the washing buffer and working buffer solution were configured (Enzo Life Sciences, USA). The other steps were briefly as follows: removed the medium and washed with the washing buffer for 3 times. Add 100ul of working buffer into each well and incubate at 25° C for 30 min away from light. Fixed at 25° C for 15 minutes and washed for 3 times. DAPI was applied to stain nucleus at 25° C and shielded from light for 15 min. The laser confocal microscope was observed and photographed at 60 power [14]. Rapamycin at a concentration of 50nM was used as a positive control.

### Differential expression analysis of autophagy-related genes in TCGA database

Autophagy related genes were downloaded from the human Autophagy database (http://www.autophagy.lu/). The R language package limma was used to extract autophagy related gene expression data from the expression profile of TCGA patients with gastric cancer. Pheatmap and limma R packages performed differential expression analysis of the above expression data and plotted them into heat map [15].

### Consensus clustering

ConsensusClusterPlus R package was used to analyze autophagy related gene expression data after the above differential expression analysis [16].

### Construction of risk score model

The glmnet and survival R package were used to construct the risk prognosis model [13, 17, 18].

### Gene set enrichment analysis

c2.cp.kegg.v7.4.symbols gmt was considered as the standard reference gene set. The top five signaling pathways were output after analysis [19–21].

### Statistical analysis

The unpaired T test will detect whether there is a significant difference between the two groups of data. *p<0.05* was considered as the standard to identify the significant difference between the two groups of data.

### Availability of supporting data

The data generated during this study are included in this article and its Supplementary Information files are available from the corresponding author on reasonable request.

### Consent for publication

All authors have read this manuscript and approved for submission.

## RESULTS

### Identification of differential expression of autophagy related genes and consensus clustering analysis

**Figure 1:**
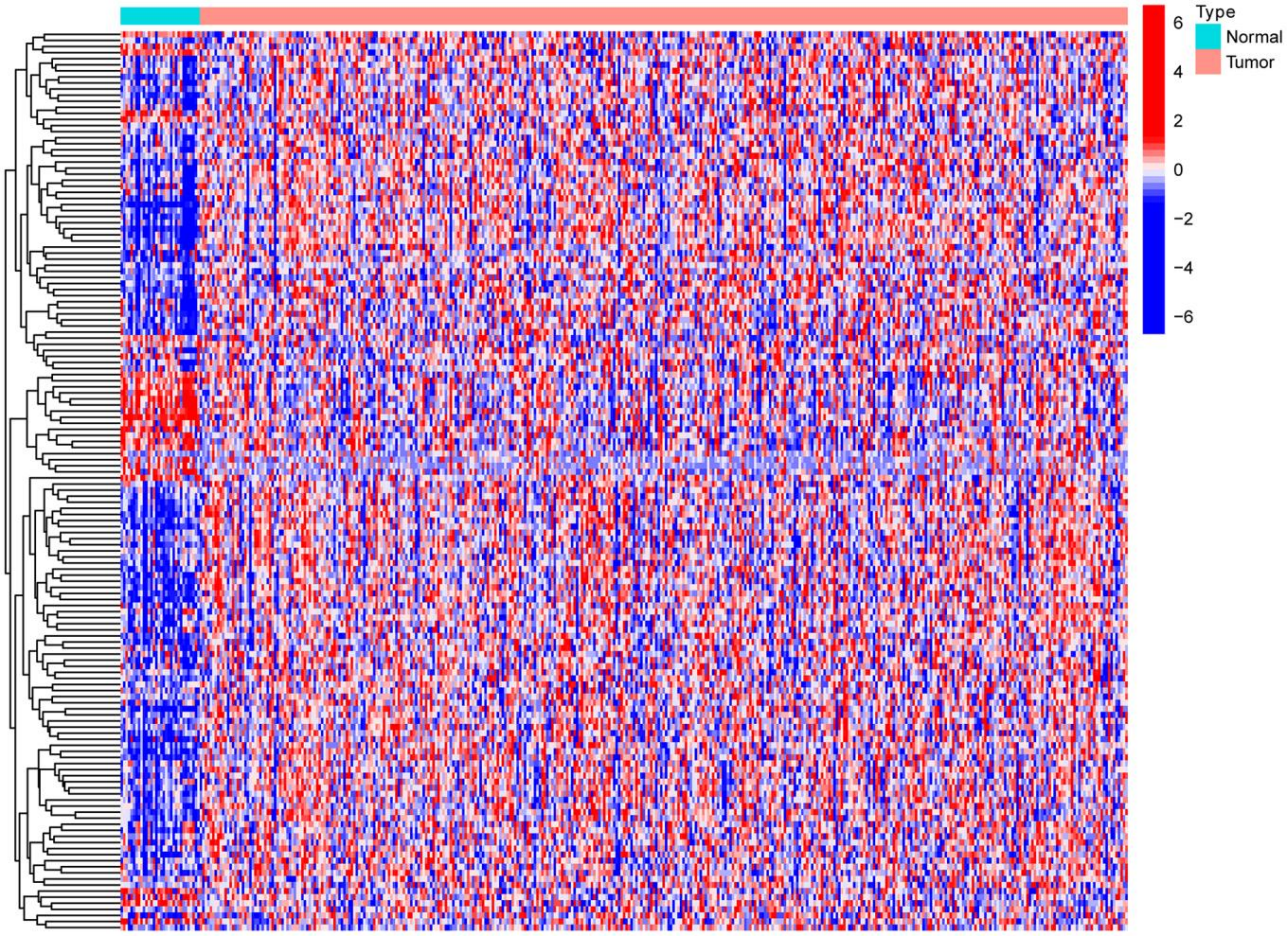
Heat map of differential expression of autophagy-related genes.

**Figure 2:**
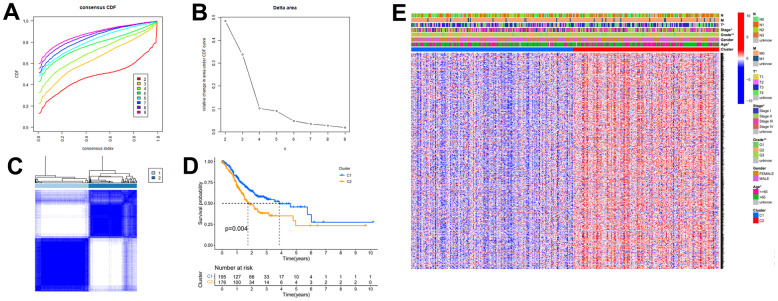
**The autophagy -related genes could classify GC into two groups by consensus clustering analysis.** (**A**) Cumulative distribution function (CDF) for k=2 to k=9. (**B**) Relative change in area under the CDF curve according to different k values. (**C**) Consensus clustering matrix of samples from TCGA dataset for k=2. (**D**) Survival analysis of patients in the C1 group and C2 group in TCGA cohort. (**E**) Heatmap of two clusters defined by the expression of autophagy-related key genes.

Gastric cancer transcriptome data were downloaded from the TCGA database, and then limma R language package was used to extract the expression profile of autophagy related genes. The limma and pheatmap R packages jointly performed differential expression analysis on the above data and plotted differential expression heat map (Figure 1). 148 genes were obtained, of which 30 were down-regulated and the rest were up-regulated. In order to obtain a more novel and larger perspective for research, consensus clustering analysis were conducted on the TCGA samples based on these 148 genes. Figure 2A shows the CDF curve change as the parameter K from 2 to 9. Figure 2B shows the area under the curve When the unsupervised clustering algorithm was used to classify the subtypes of gastric cancer patients, it was found that when the clustering k=2, the intra-group correlation was the highest and the inter-group correlation was the lowest. This indicated that two molecular subtypes were the best category selection (Figure 2C). Survival curve showed that the OS of C2 was lower than that of C1 (*p=0.004*) (Figure 2D). Finally, we combined clinicopathological parameters to draw clinical heat map after typing. As shown in Figure 2E, C1 and C2 subtype had significant differences in T, stage, grade and age, but no significant differences in other parameters such as N, M and gender.

### Build the risk prognostic model

**Figure 3:**
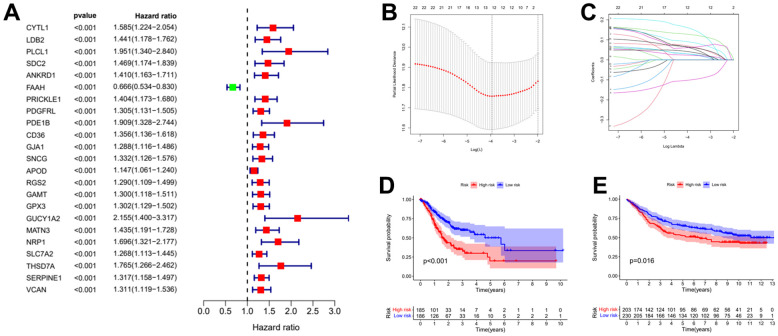
**Establishment of risk prognostic model.** (**A**) Univariate Cox regression analysis preliminary screening results. (**B**) Partial likelihood deviance was plotted versus log (Lambda). The vertical dotted line indicates the lambda value with the minimum error and the largest lambda value. (**C**) LASSO coefficient profiles of the genes screening by univariate Cox regression analysis. (**D**) The patient samples from TCGA were divided into high and low risk groups based on risk score and the OS of the groups were analyzed using Kaplan-Meier. (**E**) OS analysis of high and low risk groups from the GEO samples. LASSO: least absolute shrinkage and selection operator. OS: overall survival.

To obtain the prognostic value of autophagy related genes after subtyping, we constructed a risk score model. Firstly, the univariate Cox regression analysis was used to process the data to obtain a more accurate study scope (Figure 3A). As a result, we obtained 23 preliminarily screened genes (Table 1). Then the clinical and expression integration data of these 23 genes were put into LASSO regression analysis. We obtained a prognostic model with 12 risk genes, namely CYTL1, ANKRD1, FAAH, GJA1, SNCG, APOD, RGS2, GAMT, MATN3, NRP1, SLC7A2, and SERPINE1 (Figure 3B, 3C). Among the 12 risk genes, FAAH was negatively correlated with overall survival (OS), while the other 11 genes were positively correlated with OS (Supplementary Figure 1).

**Table 1:** Results of the consensus clustering analysis genes in the Univariate Cox regression analysis.

**Univariate analysis**				
**ID**	**HR**	**HR.95L**	**HR.95H**	**Pvalue**
CYTL1	1.58534	1.223782	2.053719	**0.000485**
LDB2	1.440534	1.177839	1.761818	**0.00038**
PLCL1	1.951046	1.34046	2.839757	**0.000483**
SDC2	1.469399	1.174088	1.838987	**0.000774**
ANKRD1	1.410378	1.162822	1.710637	**0.00048**
FAAH	0.665578	0.533716	0.830017	**0.000302**
PRICKLE1	1.404083	1.173265	1.680311	**0.000212**
PDGFRL	1.30468	1.131176	1.504797	**0.000259**
PDE1B	1.90916	1.328098	2.744446	**0.000479**
CD36	1.355607	1.136116	1.617502	**0.000735**
GJA1	1.287696	1.115884	1.485961	**0.000539**
SNCG	1.331763	1.125529	1.575786	**0.000845**
APOD	1.146886	1.060935	1.239801	**0.000564**
RGS2	1.289583	1.109078	1.499466	**0.000948**
GAMT	1.299673	1.117632	1.511364	**0.000663**
GPX3	1.301922	1.128553	1.501925	**0.000296**
GUCY1A2	2.155227	1.400157	3.317486	**0.000484**
MATN3	1.434623	1.191208	1.727778	**0.000142**
NRP1	1.69609	1.321444	2.176955	**3.34E-05**
SLC7A2	1.267743	1.112514	1.444631	**0.000371**
THSD7A	1.765319	1.265645	2.462264	**0.000815**
SERPINE1	1.316917	1.158388	1.497141	**2.59E-05**
VCAN	1.31134	1.11942	1.536164	**0.000787**

Bold words mean P value is less than 0.05; HR, hazard ratio; L, low; H, high.

**Figure 4:**
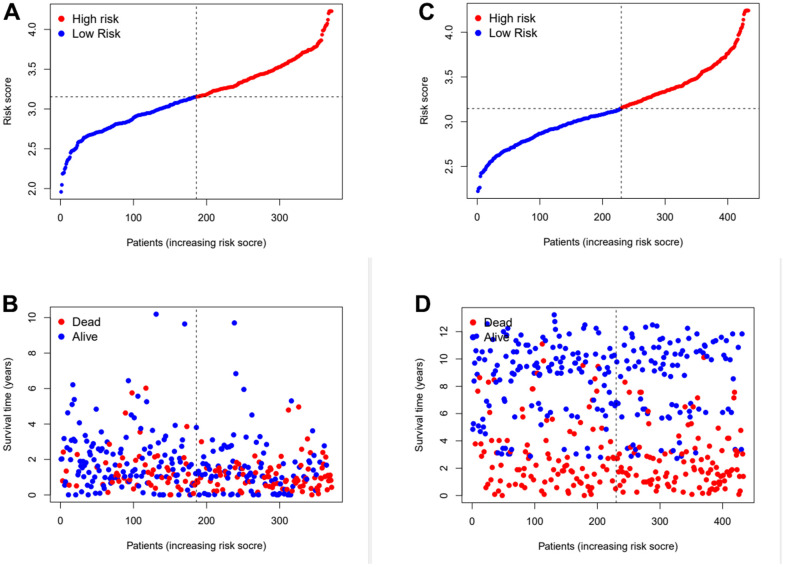
**Assess the ability of risk scores to differentiate patients’ survival status.** (**A**, **B**) The distributions of risk scores and OS status in TCGA. (**C**, **D**) The distributions of risk scores and OS status in GEO.

Subsequently, the risk coefficients of these 12 genes were used to calculate the risk scores of gastric cancer samples, and the samples were grouped according to the risk scores. The risk score is calculated with the formular: (expression of CYTL1 x 0.061+expression of ANKRD1 x 0.158+expression of FAAH x -0.101+expression of GJA1 x 0.028+expression of SNCG x 0.029+expression of APOD x 0.023 +expression of RGS2 x 0.04+expression of GAMT x 0.068+expression of MATN3 x 0.089+expression of NRP1 x 0.061+expression of SLC7A2 x 0.064+expression of SERPINE1 x 0.127. Kaplan-Meier survival curve analysis of TCGA cohorts showed that the significant difference of OS in the high-risk group was lower than that in the low-risk group (Figure 3D, *p<0.001*). The Kaplan-Meier survival curve analysis result of data set GSE88437 were in good agreement with the TCGA result (Figure 3E, *p=0.016*). Figure 4A, 4B show the survival status of patients in the high-low risk group distributed with risk scores in TCGA cohorts. These results suggest that the number of patients who die increases when the risk score exceeds the average score. Figure 4C, 4D show the distribution of patients in GSE88437 cohorts. The data show that the number of patients who have died increases with the increase of risk score. This indicated that this risk model can predict the survival status of patients to some extent.

### Assess the risk prognosis model

**Figure 5:**
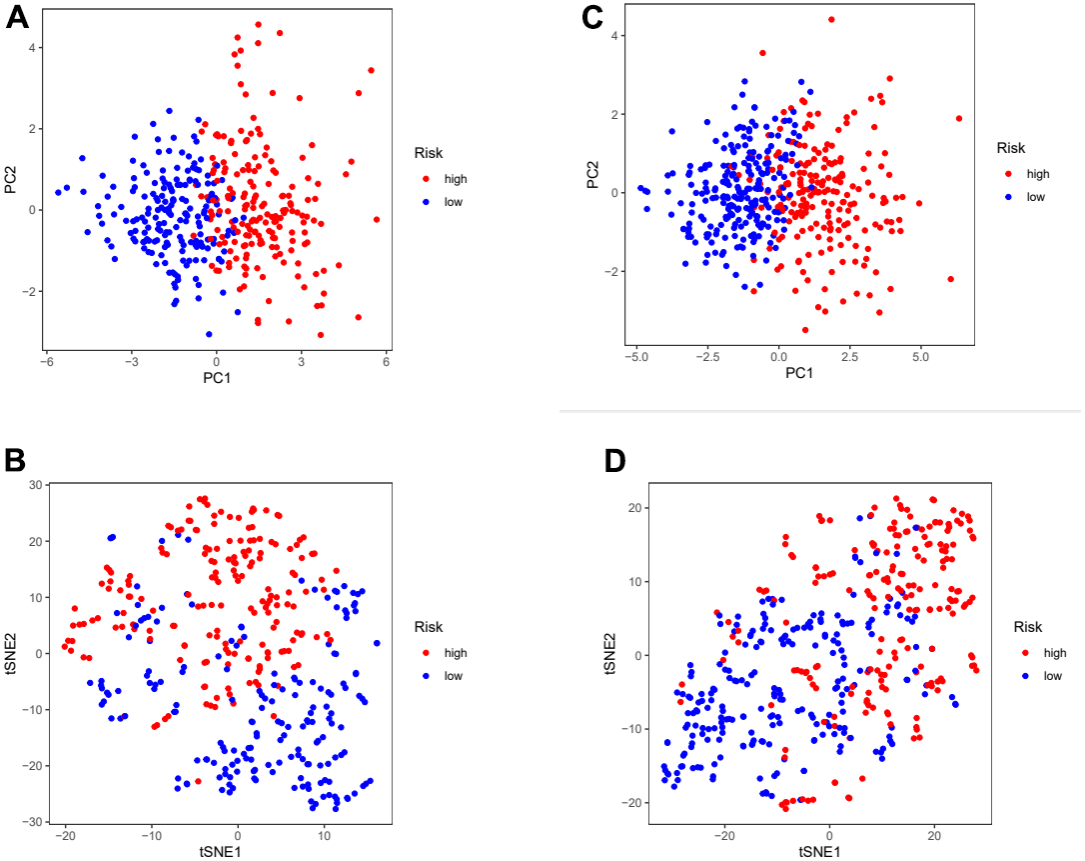
**Assess the ability of risk scores to differentiate patients in high and low risk group.** (**A**, **B**) PCA and tSNE assessed the ability of risk scores to distinguish between high and low risk groups of patients in the TCGA cohorts. (**C**, **D**) PCA and tSNE assessed the ability of risk scores to distinguish between high and low risk groups of patients in the GEOcohorts. PCA: principal components analysis; tSNE: t-distributed stochastic neighbor embedding.

**Figure 6:**
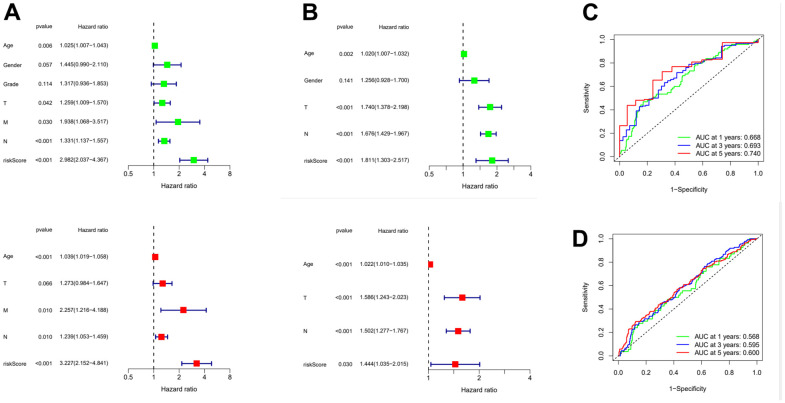
**Evaluation of risk model and time-dependent ROC curve analysis.** (**A**) Univariate and Multivariate Cox analysis of risk score and clinical characteristics in TCGA. (**B**) Univariate and Multivariate Cox analysis of risk score and clinical characteristics in GEO. (**C**) ROC curve analysis in TCGA. (**D**) ROC curve analysis in GEO. ROC: Receiver operating characteristic curve.

**Figure 7:**
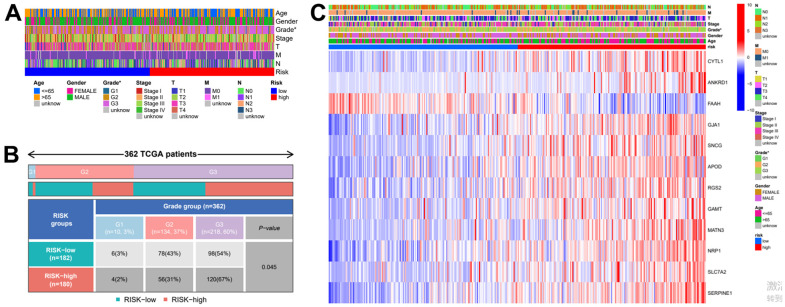
**Clinical heatmap of risk scores and risk genes.** (**A**) Clinical heat map of clinicopathological parameters and risk scores. (**B**) Clinical heat map of grade and risk scores. (**C**) Clinical heat map of risk scores, risk gene expression and clinicopathological parameters.

In order to further verify the accuracy of this risk model, we conducted the following analysis to verify it. We first apply PCA and tSNE to analyze whether risk scores can effectively evaluate patient samples. The results of TCGA cohort show that the cases were dispersed clearly, which indicates that the risk score can effectively distinguish these cases (Figure 5A, 5B). Similar results were obtained for validation group data set (Figure 5C, 5D). In the next step, Cox regression analysis combined with clinicopathological data was used to validate the risk score predictive ability of this risk model. The results in Figure 6A and Table 2 show that the risk score was found to be a valid risk prognostic option in the TCGA cohort after both univariate and multivariate Cox regression analyses. Data obtained in Figure 6B and Table 3 support the above view. The ROC was used to evaluate the accuracy and specificity of the predictive value of this model at 1, 3 and 5 years. In the ROC curve constructed by TCGA and GEO data, the AUC values were all >. 0.5 (Figure 6C, 6D). In view of these findings, we intended to delineate the predictive ability of the risk score in more detail, we constructed a risk score clinical heat map by combining the risk score with clinical characteristics. Risk scores were significantly different in Grade staging (Figure 7A). Based on this, we combined the Grade staging data to delineate the risk score in more detail (Figure 7B). The low risk group consisted of 6, 78 and 98 patients in G1, G2 and G3 respectively. The high risk group consisted of 4, 56 and 120 patients in G1, G2 and G3 respectively. Finally, we combined the expression data of the 12 risk genes to create a clinical heat map of risk gene expression (Figure 7C).

**Table 2:** Results of the risk score and clinical characteristics in the univariate and multivariate Cox regression analysis.

**Univariate analysis**					**Multivariate analysis**			
**Parameter**	**HR**	**HR.95L**	**HR.95H**	**Pvalue**	**HR**	**HR.95L**	**HR.95H**	**Pvalue**
Age	1.024932	1.007256	1.042918	**0.00552813**	1.038602	1.019385	1.05818	7.04E-05
Gender	1.445034	0.989755	2.109736	0.056568327				
Grade	1.31705	0.936361	1.852513	0.11360544				
T	1.258574	1.008851	1.570111	**0.041544534**	1.273273	0.984186	1.647272	0.065967
M	1.937808	1.067767	3.516777	**0.029585402**	2.256715	1.216061	4.187917	**0.009878**
N	1.33078	1.137148	1.557382	**0.00036809**	1.239293	1.05266	1.459015	**0.009989**
riskScore	2.982456	2.03694	4.366867	**1.94E-08**	3.227462	2.151736	4.84098	**1.48E-08**

Bold words mean P value is less than 0.05; HR, hazard ratio; L, low; H, high.

**Table 3:** Results of the risk score and clinical characteristics in the Univariate and Multivariate Cox regression analysis.

**Univariate analysis**					**Multivariate analysis**			
**Parameter**	**HR**	**HR.95L**	**HR.95H**	**Pvalue**	**HR**	**HR.95L**	**HR.95H**	**Pvalue**
Age	1.019603	1.006985	1.032379	**0.00224636**	1.022283	1.009966	1.034749	**0.000366**
Gender	1.255635	0.927537	1.699792	0.140706896				
T	1.740416	1.37793	2.19826	**3.31E-06**	1.58594	1.243204	2.023164	**0.000205**
N	1.676345	1.428609	1.967042	**2.42E-10**	1.502202	1.277423	1.766535	**8.63E-07**
riskScore	1.810884	1.302967	2.516793	**0.000406661**	1.444134	1.035238	2.014536	**3.05E-02**

Bold words mean P value is less than 0.05; HR, hazard ratio; L, low; H, high.

### Gene set enrichment analysis

**Figure 8:**
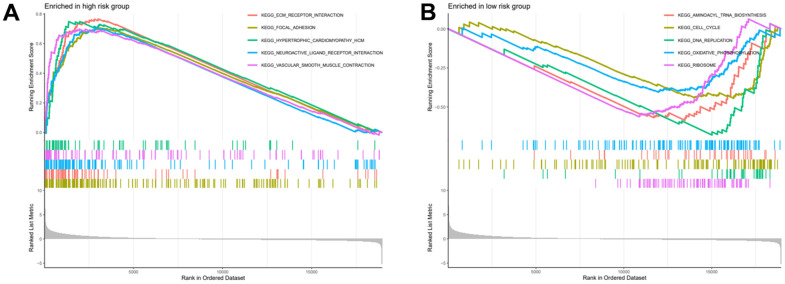
**High and low risk group gene set enrichment analysis.** (**A**) Gene set enrichment analysis for high risk group. (**B**) Gene set enrichment analysis for low risk group.

After risk score grouping, a gene set enrichment analysis was performed to provide better understanding of the pathogenesis of the different subgroups of patients. The signalling pathways enriched in the high-risk group were: Ecm Receptor Interaction, Focal Adhesion, Hypertrophic Cardiomyopathy Hcm, Neuroactive Ligand Receptor Interaction, Vascular Smooth Muscle Contraction (Figure 8A). The signalling pathways enriched in the low-risk group were: Aminoacyl Trna Biosynthesis, Cell Cycle, DNA Replication, Oxidative Phosphorylation, Ribosome (Figure 8B).

### NRP1 possesses the ability to regulate the autophagy and proliferation of gastric cancer cells through the Wnt/β-catenin signaling pathway

**Figure 9:**
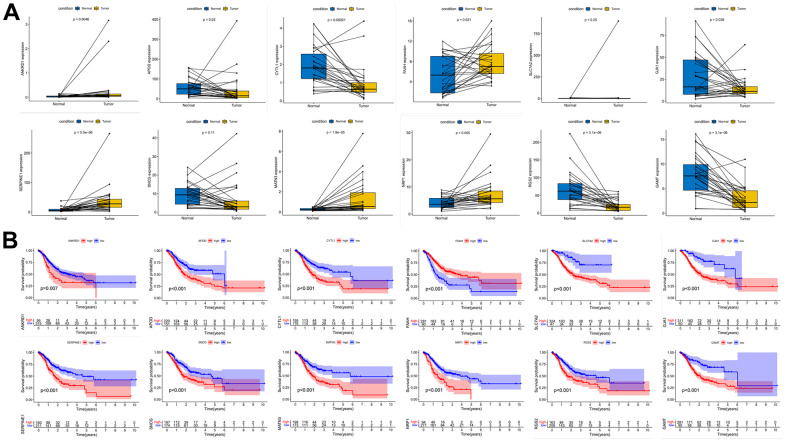
**Paired expression analysis and survival curve analysis of risk genes.** (**A**) Risk gene expression pair analysis. (**B**) Risk gene survival curve analysis.

**Figure 10:**
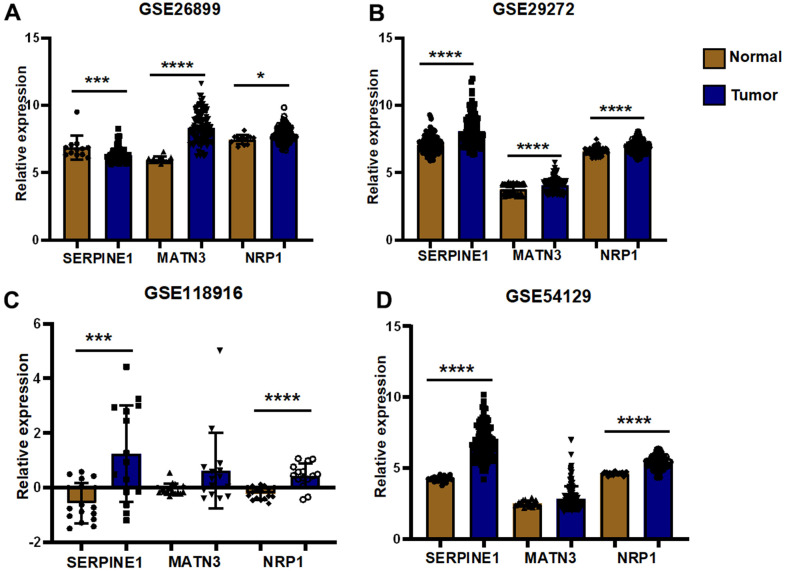
**External validation of GEO datasets.** (**A**) Differential expression analysis was performed in GSE26899 (**B**) Differential expression analysis was performed in GSE29272 (**C**) Differential expression analysis was performed in GSE118916 (**D**) Differential expression analysis was performed in GSE54129(*P< 0.05; **P< 0.01; ***P< 0.001;****P< 0.0001).

We performed expression pairwise analysis of 12 risk genes and survival curve analysis, at which point we found that MATN3, NRP1 and SERPINE1 were highly expressed in the tumour tissue and their high expression group had significantly lower survival rates, while the rest of the genes had conflicting expression data and survival analysis results (Figure 9A, 9B). For more precise study of specific gene, we selected four gastric cancer expression datasets from the GEO database for screening (GSE26899, GSE29272, GSE118916 and GSE54129). The results in Figure 10 show that MATN3 was not significantly differentially expressed in the datasets GSE118916 and GSE54129 (Figure 10C, 10D), while SERPINE1 was highly expressed in normal tissues in the GSE26899 dataset (Figure 10A, 10B), so we finally selected NRP1 as the final study target.

**Figure 11:**
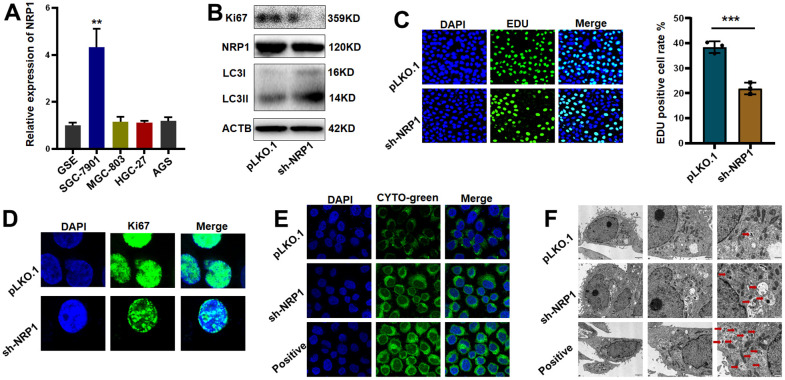
**Validation of NRP1’s regulation of tumor autophagy and proliferative capacity *in vitro*.** (**A**) QPCR was used to detect NRP1 gene expression in gastric cancer cell lines. (**B**) western blot analysis. (**C**, **D**) EdU assay and cell immunofluorescence were used to detect the effect of NRP1 knockout on cell proliferation. (**E**) Autophagy assay to detect the effect of NRP1 knockdown on autophagy. (**F**) The effect of NRP1 knockdown on autophagy was detected by transmission electron microscopy. (*P< 0.05; **P< 0.01; ***P< 0.001;****P< 0.0001).

Differential expression detection of NRP1 by QPCR was performed (Figure 11A). Next, we constructed NRP1 stable knockdown cell line and applied western blot to detect the expression levels of NRP1, the cell proliferation protein marker Ki67 and the cell autophagy-related marker LC3I/II. As shown in Figure 11B and Supplementary Figure 2A, the expression of Ki67 decreased but the ratio of LC3II to LC3I was increased when NRP1 was knocked down, suggesting that knockdown of NRP1 may cause a decrease in cell proliferation and an upregulation of autophagy. Following this lead, we performed EdU experiment and cellular autophagy labeling assay. As shown in Figure 11C, 11D, the proliferative capacity of cells decreased by about 20% when NRP1 was knocked down, and the intensity of cellular autophagy increased (Figure 11E). To provide more reliable evidence that knockdown of NRP1 causes cellular autophagy, we fixed the treated cells and applied transmission electron microscopy to photograph the number and morphological changes of autophagic vesicles. It can be clearly observed in Figure 11F that the number of intracellular autophagic vesicles increased when NRP1 was knocked down.

**Figure 12:**
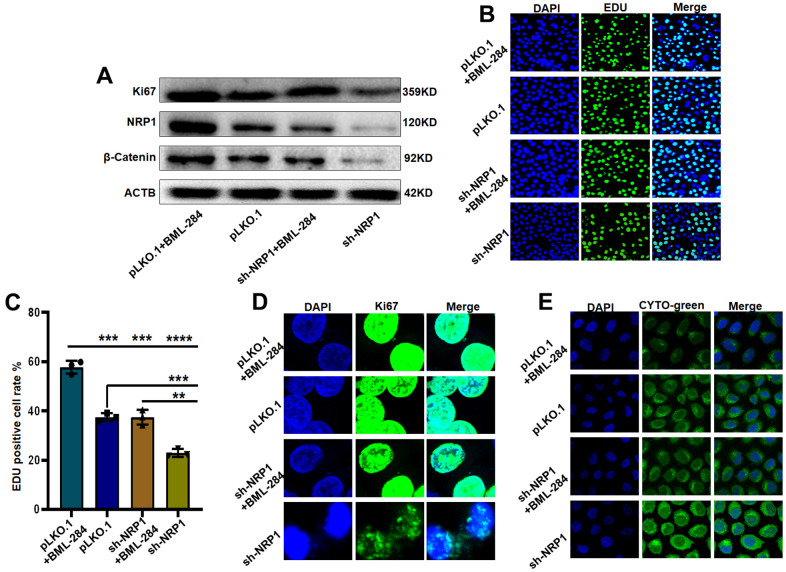
**NRP1 regulates tumour autophagy and proliferation through the Wnt/β-catenin pathway.** (**A**) Western blot detection of relevant signaling pathways. (**B**, **C**) EdU was used to detect changes in cell proliferation ability under different treatments. (**D**) cellular immunofluorescence were used to detect changes in cell proliferation ability under different treatments. (**E**) Detection of autophagy under different group treatments. (*P< 0.05; **P< 0.01; ***P< 0.001;****P< 0.0001).

Next, we explore the signaling pathway through which NRP1 exerts its biological function. Western blot results showed that the level of β-Catenin decreased when NRP1 knockdown was achieved. However, when activator BML-284 was added to the knockdown cells, the expression of β-Catenin was elevated and the expression of Ki67 was increased with the addition of BML-284 (Figure 12A and Supplementary Figure 2B). Subsequent EdU and Cellular immunofluorescence assays provided further effective evidences (Figure 12B, 12C). EdU results showed that when BML-284 was added, the proliferation capacity of NRP1 knockdown group increased by 19.5%, while that of control group increased by 21.43%. The results of the cellular immunofluorescence assay were similar to those of the EdU assay ((Figure 12D). Finally the autophagy assay showed that autophagy was inhibited when BML-284 was added to the cells (Figure 12E). Taken together, the above experimental results suggest that NRP1 may exercise its ability to regulate autophagy and cell proliferation through the Wnt/β-Catenin signalling pathway.

**Figure 13:**
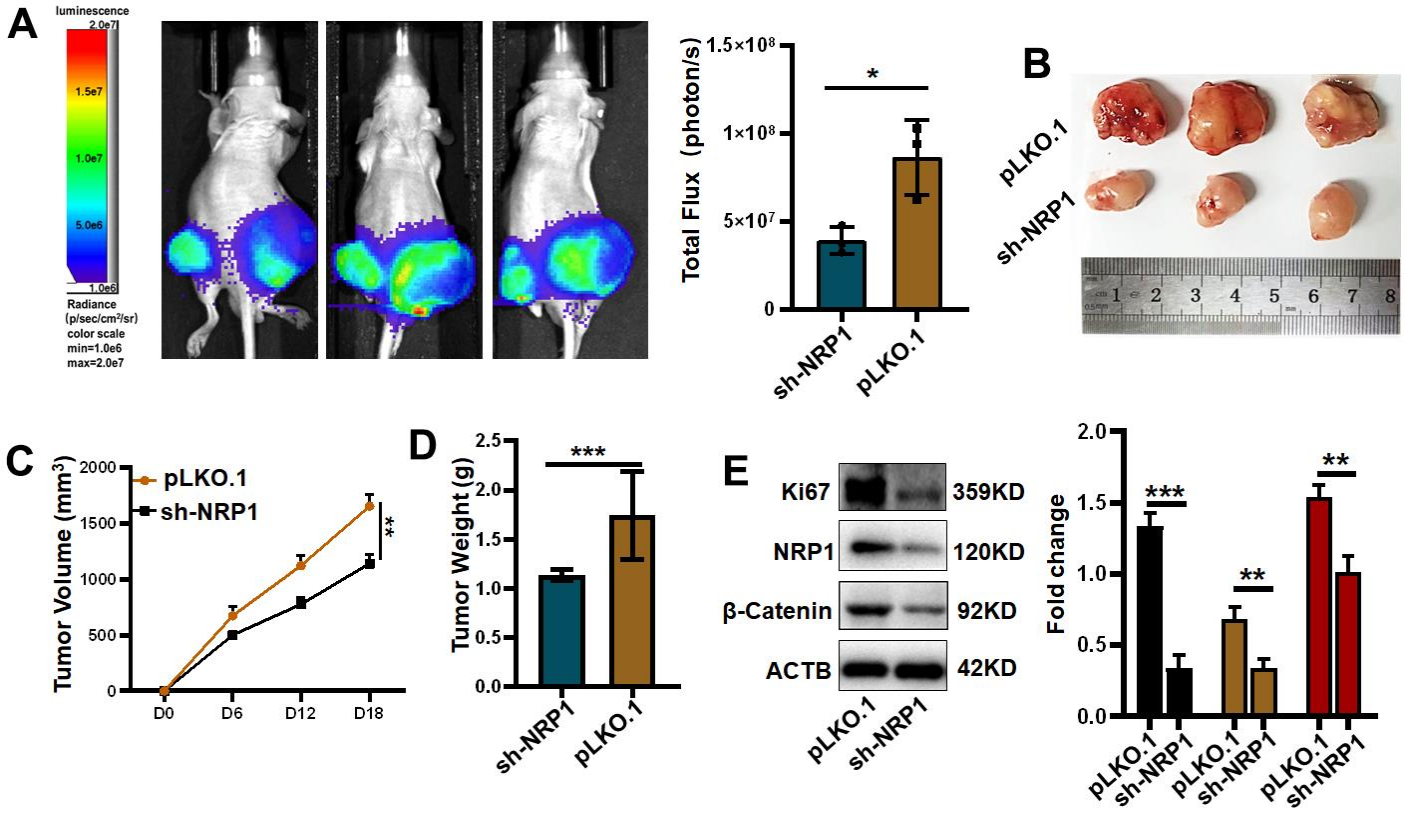
**Verifying the ability of NRP1 to regulate tumors *in vivo*.** (**A**) Detection of xenografts by animal imaging. (**B**) Image of xenograft tumor. (**C**) Tumor volume growth curve. (**D**) Tumor weight. (**E**) Western blot protein detection and quantitative analysis. (*P< 0.05; **P< 0.01; ***P< 0.001;****P< 0.0001).

Finally, in order to investigate whether NRP1 affects tumour growth *in vivo*, a nude mouse xenograft tumour model was constructed for this purpose. The xenograft tumour models were examined in the Spectral Instruments Imaging System on day 18 after cells inoculation. The results showed a 54.68% decrease in bioluminescence signal in the sh-NRP1 knockdown group. The average decrease in xenograft tumour volume was 512.7 mm^3^, which represents a 30.97% tumour suppression rate due to knockdown of NRP1, and an average decrease in xenograft tumour weight of 47.34% to approximately 0.98 g (Figure 13B–13D). Finally, we extracted the proteins from the xenograft tumors and performed western-blot analysis. As shown in the Figure 13E, the expressions of Ki67, NRP1 and β-Catenin were significantly decreased in the NRP1 knockdown group compared with the control group.

## DISCUSSION

Gastric cancer is a commonly diagnosed cancer of the digestive system However, the pathogenesis and treatment of gastric cancer has been unsatisfactory. Autophagy is used as a cellular stress mechanism in response to adverse circumstances such as lack of nutrients, oxidative stress or organelle damage. Although most previous studies have reported that cellular autophagy is a self-protective mechanism to prevent cancer development, cellular autophagy can also perform proto-oncogenic functions to promote cancer development. For example, cancer metastasis occurs in a number of steps, including angiogenesis, alteration of the tumour microenvironment, changes in the extracellular matrix and the Epithelial-mesenchymal transition (EMT) [22, 23]. Cellular autophagy can both promote tumour metastasis in this process and stop it by degrading key proteins. Given the important regulatory role of cellular autophagy in cancer, this study will focus on autophagy-related genes and their molecular regulatory mechanisms in gastric cancer.

In our research, we applied autophagy-related genes to stratify gastric cancer cohorts in the TCGA database by consensus clustering analysis. The samples were found to be most appropriately divided into two clusters, and overall survival was lower for the C2 group than for the C1 group. We then constructed risk-prognosis models based on the differential genes after subtype stratification. The model contained 12 prognostic genes, of which FAAH was negatively associated with OS and the remaining genes were positively associated with OS. CYTL1 is a secreted protein involved as a cytokine in the regulation of some cancers and cellular inflammation [24–26]. In neuroblastoma, high expression of CYTL1 and its knockdown causes a decrease in cell proliferation and migration [27]. In other cancers, however, CYTL1 exhibits the opposite effect, acting as a tumour suppressor and inhibiting tumourigenesis. Notably, similar to previous studies, CYTL1 functions as a prognostic factor in gastric cancer [28]. ANKRD1 is a universal tumor suppressor that plays a role in many cancers through positive regulation of apoptosis, such as pancreatic and lung cancers [29–32]. However, it can also play a role in promoting cancer. For example, ANKRD1 is highly expressed in ovarian cancer and will increase the sensitivity of ovarian cancer cells to cisplatin after knockdown to enhance the therapeutic effect [33]. Therefore, the role of ANKRD1 in different cancers should be analyzed according to the actual situation of cancer. NRP1 is involved in regulating the occurrence, drug resistance and metastasis of bladder cancer, lung cancer, stomach cancer, breast cancer, ovarian cancer and other cancers [34–37]. Although NRP1 has been reported to be associated with poor prognosis and metastasis in gastric cancer patients, little is known about its role in regulating autophagy in gastric cancer. The results of our bioinformatics analysis were consistent with previous reports. Patients with high expression of NRP1 had poor survival and the results of gene set enrichment analysis also showed that the high-risk group was highly correlated with tumor metastasis, which is consistent with other reports that NRP1 is involved in regulating the metastasis of various tumors [38–41]. *In vitro*, we found that NRP1 promoted the proliferation but inhibited autophagy of gastric cancer cells, which was consistent with the previous report in colon cancer [42]. As it has been reported in the literature that Wnt signaling pathway is closely related to the regulation of cellular autophagy. In glioblastoma, multiple myeloma, activation of Wnt signaling pathway inhibits cellular autophagy, while other reports suggest that activation of Wnt signaling pathway promotes cellular autophagy [43–48]. Therefore, the regulatory relationship between the Wnt signaling pathway and autophagy in gastric cancer should be explored through research. Subsequently, we conducted a study on which signaling pathway NRP1 plays its role in gastric cancer. The results showed that when the Wnt signaling pathway activator BML-284 was added to the cells knocking down NRP1, the autophagy in the cells decreased, but the cell proliferation ability increased significantly. This suggests that NRP1 may regulate cell autophagy and proliferation through the Wnt signaling pathway. In conclusion, we found that NRP1 can promote the proliferation of gastric cancer cells and inhibit the autophagy of gastric cancer cells. However, there are still some defects in our research. For example, there are no clinical samples to verify the experimental results and more detailed pathological mechanism studies have not been carried out. Further elucidation of the interaction of NRP1 with autophagy and Wnt signaling pathways in gastric cancer is the focus of our next study.

In summary, a risk-prognosis model for 12 autophagy-related genes was obtained and the ability of this model to differentiate patient groups and prognosis was validated. After screening and validation, we found that the risk gene NRP1 possesses the competence to regulate cell proliferation and autophagy, and the data we obtained suggest that NRP1 may be a promising focus for the development of new therapeutic agents or gene therapy targets.

## Supplementary Material

Supplementary Figures
