# Supplementary material for: NRP1 regulates autophagy and proliferation of gastric cancer through Wnt/β-catenin signaling pathway
Source: Aging (Albany NY). 2023 Mar 7;15(17):8613–29. doi: 10.18632/aging.204560 (PMC10522364; doi:10.18632/aging.204560)
Supplement: Supplementary Figures [file aging-15-204560-s001.pdf]

SUPPLEMENTARY FIGURES

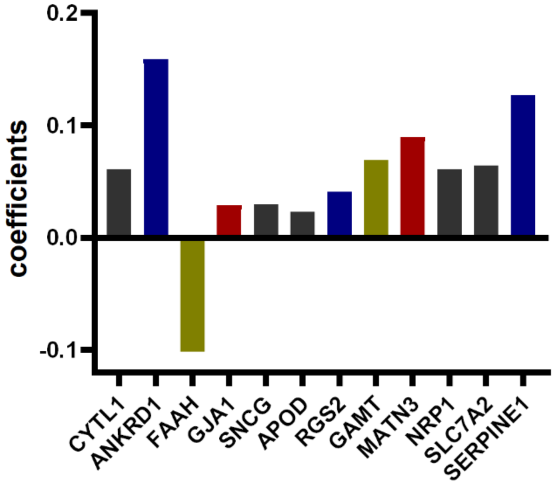

Supplementary Figure 1. The 12 genes were selected by least absolute shrinkage and selection operator. (LASSO) Cox analysis in TCGA dataset and histogram of coefficient.

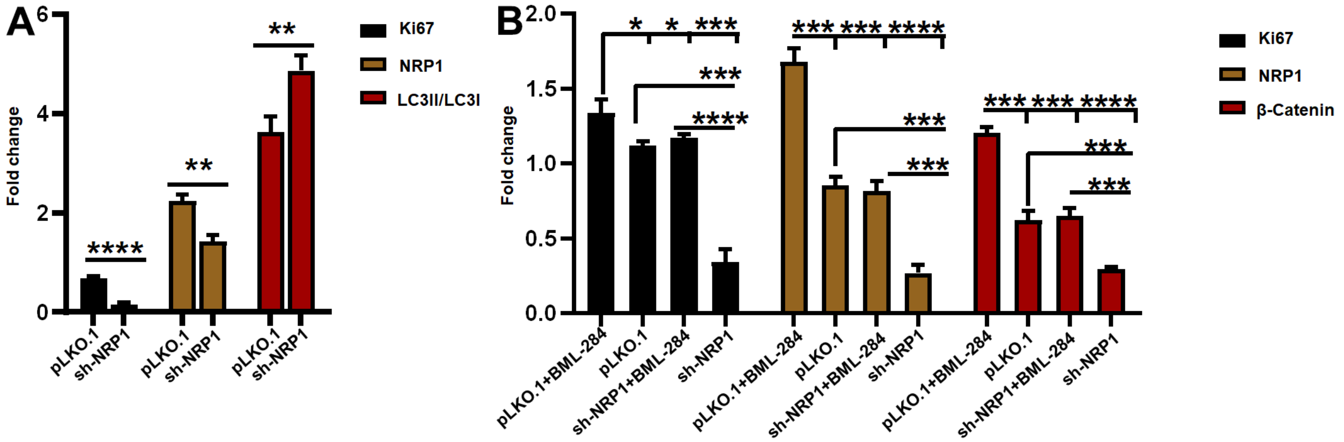

Supplementary Figure 2. Quantitative analysis of protein expression. (**A**) Quantitative analysis of protein expression after knockdown of NRP1. (**B**) Quantitative analysis of protein expression under different treatments. (**P*< 0.05; ***P*< 0.01; ****P*< 0.001; *****P*< 0.0001).
